# Supplementary material for: Moesin (MSN) as a Novel Proteome-Based Diagnostic Marker for Early Detection of Invasive Bladder Urothelial Carcinoma in Liquid-Based Cytology
Source: Cancers (Basel). 2020 Apr 21;12(4):1018. doi: 10.3390/cancers12041018 (PMC7225967; doi:10.3390/cancers12041018)
Supplement: Supplementary file 1 [file cancers-12-01018-s001.zip › cancers-764766-supplementary/cancers-764766.pdf]

cancers

Supplementary Materials: Moesin (*MSN*) as a Novel Proteome-Based Diagnostic Marker for Early Detection of Invasive Bladder Urothelial Carcinoma in Liquid-Based Cytology

Jeong Hwan Park [1,2,†], Cheol Lee [1,3,†], Dohyun Han [4,5,†], Jae Seok Lee [6,†], Kyung Min Lee [7], Min Ji Song [3], Kwangsoo Kim [4], Heonyi Lee [4,5], Kyung Chul Moon [1,3], Youngsoo Kim [8], Minsun Jung [1,3], Ji Hye Moon [1,3], Hyebin Lee [9,*], Han Suk Ryu [1,3,*]

1. Supplementary Material and Methods

1.1. Patient Selection and Clinicopathologic Review

We retrospectively reviewed all bladder urothelial carcinoma (BUC) samples surgically removed by cystoscopic biopsy, transurethral resection of bladder (TUR-B), and partial or radical cystectomy and matched liquid-based cytology (LBC) samples including voided urine and bladder washing collected between 1 January, 2013 and 31 December, 2015 at Seoul National University Hospital. Three urologic pathologists (J.H.P., K.C.M., and H.S.R.) independently reviewed surgical specimens and evaluated according to WHO classification [1-3]. Matched LBC specimens were also evaluated according to the Paris system [4,5]. LBC specimens with a diagnosis of 'suspicious for high-grade urothelial carcinoma (SHGUC)' or 'high-grade urothelial carcinoma (HGUC)' were enrolled in this study. For proteomic analysis, LBC specimens with >50% cancer cell purity were included. We retrieved cases with matched cytology and surgical specimens and grouped them into non-invasive BUC (NIBUC) (pTa/pTis), stromal-invasive BUC (SIBUC) (pT1), and muscle-invasive BUC (MIBUC) (pT2+) [6]. Finally, we selected 16 cytology samples for quantitative proteomic analysis. Among them 6 cases were NIBUC, 5 cases were SIBUC, and 5 cases were MIBUC. We also collected independent 30 LBC specimens consisting of 13 cases of NIBUC, 10 cases of SIBUC, and 7 cases of MIBUC to verify the diagnostic utility of immunocytochemistry (ICC). The criteria for both proteomic and ICC validation were as follows: (1) consistent cytologic diagnosis among three urologic pathologists (all three pathologists diagnosed SHGUC or HGUC or at least two of them diagnosed SHGUC or HGUC and the other diagnosed atypical cells according to the Paris system), (2) cellular specimen with fewer inflammatory cells (cellularity with > 50%), (3) no previous bacillus Calmette–Guérin (BCG) treatment, and (4) no neoadjuvant chemotherapy. This study was approved by the Institutional Review Board of Seoul National University Hospital in agreement with the Declaration of Helsinki (IRB No. H-1602-150-747).

1.2. Label-Free Sample Preparation for Proteomics Analysis of LBC

All urine samples were fixed with BD CytoRich™ Clear Preservative Fluid (BD Diagnostics-TriPath Imaging, Burlington, NC) and prepared using the SurePath liquid-based preparation method according to the manufacturer's instructions [7,8]. A total of 300 μL was aspirated from each sample and added to the corresponding slide.

An individual LBC sample of 13 mm diameter was scraped to collect well-preserved populations of unstained cells in individual Eppendorf tubes. Cell pellets were lysed with 100 μL of sodium dodecyl sulfate (SDS) buffer (4% SDS; 100 mm Tris, pH 7.4; and 1 mm tris(2-carboxyethyl)phosphine (TCEP)). Samples were lysed by sonication and boiling in a heat block at 95 °C for 30 min. Proteins were digested using the filter-aided sample preparation (FASP) procedure, as previously described [9]. Briefly, 50 μL of samples was mixed with 200 μL of urea solution (0.2 mL of 8 M urea in 0.1 M Tris/HCl, pH 8.5), then loaded onto a 30K spin filter (EMD Millipore, Billerica, MA). Buffer was exchanged with the urea

solution by centrifugation (140,000 x *g* for 20 min). Reduced cysteines were alkylated by adding an iodoacetamide solution and the samples were kept without light at room temperature for 30 min. An additional 50 mM ammonium bicarbonate was added three times to exchange the urea solution. Finally, proteins were digested at 37 °C overnight with sequencing-grade modified porcine trypsin (Promega, Madison, WI) at an enzyme to protein ratio of 1:100. After overnight incubation at 37 °C, the filtration unit containing peptides was transferred to new collection tubes, followed by centrifugation for 20 min and was eluted with 50 μL of 0.5 M NaCl to increase the yield of digested protein. The resultant supernatants were acidified with 1% trifluoroacetic acid (TFA).

1.3. High-pH Fractionation

Eluted peptides were desalted using in-house C18 Stage Tips, as previously described [10]. C18 Empore disk membranes (3M, Bracknell, UK) were placed into the bottom of 200 μL yellow pipette tips. POROS 20 R2 reversed-phase medium (Applied Biosystems, Foster City, CA) was dissolved in 1 mL MeOH and 100 μL of the mixture was loaded separately into the tip for two rounds of filtration with MeOH, which was followed by washing of packed microcolumns with 100 μL of MeOH and 100% acetonitrile (ACN) consecutively and equilibration three times with 100 μL of 0.1% TFA. After samples were loaded, microcolumns were washed three times with 100 μL of 0.1% TFA, and peptides were subsequently eluted with a series of elution buffers containing 40%, 60%, and 80% ACN (100 μL) in 0.1% formic acid. Finally, all eluates were dried in a vacuum centrifuge for liquid chromatography-tandem mass spectrometry (LC-MS/MS) analysis.

1.4. Liquid Chromatography-Tandem Mass Spectrometry

The fractionated peptides were loaded to perform LC-MS/MS analysis using a Q Exactive Plus Hybrid Quadrupole-Orbitrap mass spectrometer (Thermo Fisher Scientific Inc.), coupled to an Ultimate 3000 RSLC system (Dionex, Sunnyvale, CA) via a nano-electrospray source.

Peptide samples were separated on a two-column system consisting of a trap column and an analytical column (75 μm × 50 cm) with a 120 min gradient from 7% to 32% ACN at 300 nL/min and analyzed by mass spectrometry. Column temperature was maintained at 60 °C and survey scans (350 to 1650 m/z) were acquired with a resolution of 70,000 at 200 m/z. A top-20 method was used to select precursor ions with an isolation window of 1.2 m/z. MS/MS spectra were acquired at an higher-energy collisional dissociation (HCD)-normalized collision energy of 30, with a resolution of 17,500 at 200 m/z. The maximum ion injection times for the full scan and MS/MS scan were 20 and 100 ms, respectively.

1.5. Invasion and Migration Assay Using BUC Cell Lines

We purchased eight BUC cell lines from the American Type Culture Collection (ATCC; Manassas, VA, USA) for invasion and migration assessment. These included T24, J82, 253J-BV, 253J, 5637, RT4, HT1376, and HT1197 BUC cell lines. RNA was extracted using an eCube RNA Mini Kit (Philekorea Technology, Seoul, Korea). RNA yield and purity were assessed using a DS-11 spectrophotometer (Denovix Inc, DE, USA). Total RNA (300 ng) was added to the sample preparation reaction in the available 5 μL volume. RNA quality was verified using a Fragment Analyzer system (Advanced Analytical Technologies, IA, USA). The digital multiplexed nanoString nCounter human mRNA expression assay (nanoString Technologies) was performed. The mRNA data analysis was performed using the nSolver software analysis. The mRNA profiling data was normalized using housekeeping genes. Based on invasion and migration capacity, BUC cell lines were categorized as invasive BUC cell line (IBUC_CL) and non-invasive BUC cell line (NIBUC_CL).

1.6. Tandem Mass Tag Labeling-Based Sample Preparation for Proteomics Analysis of BUC Cell Linen

The tandem mass tag (TMT) reagent (0.8 mg) was dissolved in ACN, and 10 μL of the reagent was added to 30 μL of peptides along with ACN to reach a final concentration (30% *v*/*v*). The peptides were

subsequently pooled after incubation at room temperature for 2 h and desalted with an in-house C18 Stage Tip. Six batches of 6-plex TMT kits (Thermo Fisher Scientific) were used to label the six samples consisting of BUC cells with invasive features and non-invasive BUC cells. After the labeled peptides were pooled, the samples were separated using high-pH reversed-phase liquid chromatography.

1.7. Data Processing for Peptide Identification

MS/MS spectra were searched against the Human UniProt protein sequence database (December 2014, 88,657 entries) using the Andromeda search engine [11]. Primary searches were performed using a 6-ppm precursor ion tolerance for total protein level analysis. The MS/MS ion tolerance was set to 20 ppm. Cysteine carbamidomethylation was set as a fixed modification and N-acetylation of protein and oxidation of methionine were set as variable modifications. Enzyme specificity was set to full tryptic digestion with a minimum length of six amino acids and up to two missed cleavages. The false discovery rate (FDR) was set to 1% at the peptide, protein, and modification level. We enabled the 'Match between Runs' option to maximize the number of quantification events on the MaxQuant platform.

MS raw files of cell lines were processed using the Proteome Discoverer 2.1 software interfaced with the SEQUEST-HT search engine based on the Human UniProt database (December 2014, 88,657 entries), which included forward and reverse protein sequences and common contaminants as previously described [12].

1.8. Bioinformatics and Statistical Analyses

Annotated MS/MS spectra can be accessed through MS-Viewer [13] (http://msviewer.ucsf.edu/prospector/cgi-bin/mssearch.cgi?report_title=MS-viewer&search_key=d4qfuhxipu&search_name=msviewer) with the following search keys: d4qfuhxipu. Gene ontology annotation was explicated using ToppGene Suite resources (https://toppgene.cchmc.org/) [14]. Interaction network models were constructed using String [15] and illustrated using Cytoscape ver3.7.1 [16]. For the quantitative analysis of label-free quantification (LFQ) and the logarithmized intensities of the TMT reporter ions, statistical analyses were performed using the Perseus software [17]. We first filtered out proteins with at least eight quantified values in each group. For pairwise comparison of proteomes, two-sided t-tests were performed using permutation-based FDR and a significance level of 5%. The cell calculations for the invasion assay were made using Hill's equation in GraphPad Prism software 8.0.

1.9. Cell Migration and Invasion Assays with Small Interfering RNA (siRNA) Transfection

The T24 and J82 cell lines were evaluated for cell migration and invasion. Among three invasive BUC cell lines, 253J-BV was not included for further study because it was derived from the metastatic lymph node. The cells were cultured in DMEM (Gibco, CA, USA) containing 10% fetal bovine serum (FBS; Invitrogen, Carlsbad, CA, USA) and 1% penicillin/streptomycin (Gibco). Cells were maintained at 37 °C in a humidified atmosphere of 95% air and 5% CO_2, and periodically screened for mycoplasmic contamination. Quantitative cell migration and invasion were assessed using 24-well inserts (Corning Incorporated, NY, USA) with 8 μm pores, according to the manufacturer's instruction. In brief, for the transwell migration assay, transfected cells (1×10^5 cells) were seeded into the upper chamber and a medium containing 10% FBS in the lower chamber. After incubation for 24 h, the cells on the top side of the membrane were removed entirely using a cotton swab. The remaining migrated cells were washed with PBS, fixed in 4% paraformaldehyde, and stained with 1% crystal violet for 10 min. Then, the migrated cells were imaged and counted on three randomly selected fields under a microscope (Nikon, Tokyo, Japan). The experiments were performed in triplicate. For the in vitro invasion assay, the upper wells of the Boyden chambers were coated with 2 mg/mL of Matrigel (Corning) at 37 °C in a

5% CO_2 incubator for 2 h. The cells (2 × 10^5 cells) were seeded into the upper chamber and a medium containing 10% FBS in the lower chamber. The rest of the assay was performed as described above.

RNA interference siRNAs targeting moesin (*MSN*) and an AccuTarget Negative Control siRNA were purchased from Bioneer (Daejeon, Korea). The sequences for *MSN* were as follows: si*MSN*-1, 5′-GUCGCAAGCCUGAUACCAU-3′; si*MSN*-2, 5′-CAGAUCGAGGAACAGACUA-3′. Cells were transfected using Lipofectamine RNAiMAX (Invitrogen) following the manufacturer's instruction. After incubation for 48 h, *MSN* gene silencing was confirmed by assessing mRNA expression levels. Other siRNAs targeting *GRHL2*, *LLGL2*, *NCAM2*, and *VAPA* and their AccuTarget Negative Control siRNAs were also purchased from Bioneer (Daejeon, Korea). Their sequences were as follows: si*GRHL2*-1, 5'-GAAUGAAGAGGCGAAGAUU-3'; si*GRHL2*-2, 5'-CAGGAAAAGCGGAGCAAGU-3'; si*LLGL2*-1, 5'-CACUCUGACGGCAGCUACU-3'; si*LLGL2*-2, 5'-CAGAUCCUGAUCGGCUACA-3'; si*NCAM2*-1, 5'-CACGUUCACUGAAGGCGAU-3'; si*NCAM2*-2, 5'-CAGAAUUGGAGGGCAUCAA-3'; si*VAPA*-1, 5'-GACAGGUUCAAUUAGCUCA-3'; si*VAPA*-2, 5'-CAUCAAUUGCUAGGAACAU-3'. Transfection, incubation, and confirmation of silencing were performed as described above.

1.10. Tumor Spheroid and Three-Dimensional Spheroid Invasion Assay

Generation of tumor spheroids was processed for suspension culture using plates coated with poly-HEMA (Sigma Chemical Co., St. Louis, MO, USA). Briefly, the anti-adhesive polymer poly-HEMA (poly(2-hydroxyethyl methacrylate)) was dissolved in ethanol to a final concentration of 10 mg/mL and coated onto 60 mm culture dishes. The ethanol was evaporated overnight at room temperature, and plates were sterilized under UV light. Transfected cells were allowed to generate spheroids for 24 h onto poly-HEMA dishes, and spheroids measuring less than 70 μm in size were selected using a cell strainer (SPL life science, Pocheonsi, Korea) before being embedded into mixed collagen/matrigel matrices coating 8-well glass chamber slides (Nunc Lab-Tek, Rochester, NY, USA) after 24 h. Mixed collagen/Matrigel matrices were prepared as previously described [18]. On the next day, spheroid dissemination was observed under a phase-contrast microscope, and the samples were prepared for confocal microscopy images.

1.11. F-Actin Staining and Confocal Microscopy

3D gels were fixed with 4% paraformaldehyde, permeabilized with 0.2% Triton X-100 in PBS for 2 h of incubation, and blocked with 10% normal goat serum (Thermo Fisher Scientific, Waltham, MA, USA) for 2 h. For visualization of the actin cytoskeleton, the 3D spheroid cells were with phalloidin–rhodamine (1:100 in PBS) overnight at 4 °C. Hoechst 33342 for staining nuclei was used at 1:1000 in PBS. The stained F-actin was detected with a confocal laser scanning microscope (Leica TCS SP8; Leica microsystems, Germany).

1.12. Immunocytochemical Analysis for Assessment of the Diagnostic Utility of Invasive Marker

We assessed the diagnostic utility of an invasive marker (moesin) using independent LBC specimens. We used 30 independent LBC samples with a diagnosis of SHGUC or HGUC and with a matched histologic diagnosis of NIBUC, SIBUC, or MIBUC. ICC interpretation was evaluated as negative for no expression in tumor cells with SHGUC and HGUC and positive for the presence of expression in any tumor cell with any intensity [19]. We assessed the proportion and intensity of positive tumor cells for H-score assessment [20]. For statistical evaluation of H-score in BUC groups, we employed Kruskal–Wallis test and Mann–Whitney *U* test with the GraphPad Prism 8.0 program (GraphPad Software, Inc., CA, USA). Additionally, for cross-tabulation analysis of categorical variables as negative and positive immunostaining of moesin, we employed Pearson's χ^2 test and Fisher exact test with IBM SPSS Statistics version 20 (IBM SPSS, IL, USA). In all statistical analyses, a 2-tailed p-value < 0.05 was considered as statistically significant.

References

1. Grignon, D.J.; Al-Ahmadie, H.; Algaba, F.; Amin, M.B.; Compérat, E.; Dyrskjøt, L.; Epstein, J.I.; Hansel D.E.; Knüchel, R.; Lloreta, J.; et al. Urothelial tumours: Infiltrating urothelial carcinoma. In *WHO Classification of Tumours of the Urinary System and Male Genital Organs*, 4th ed.; Moch, H.; Humphrey, P.A.; Ulbright, T.M.; Reuter, V.E., Eds.; International Agency for Research on Cancer: Lyon, France, 2016; pp. 81–98.
2. Humphrey, P.A.; Moch, H.; Cubilla, A.L.; Ulbright, T.M.; Reuter, V.E. The 2016 WHO Classification of Tumours of the Urinary System and Male Genital Organs-Part B: Prostate and Bladder Tumours. *Eur. Urol.* **2016**, *70*, 106–119.
3. Compérat, E.M.; Burger, M.; Gontero, P.; Mostafid, A.H.; Palou, J.; Rouprêt, M.; van Rhijn, B.W.G.; Shariat, S.F.; Sylvester, R.J.; Zigeuner, R.; et al. Grading of Urothelial Carcinoma and The New "World Health Organisation Classification of Tumours of the Urinary System and Male Genital Organs 2016". *Eur. Urol. Focus.* **2019**, *5*, 457–466.
4. Barkan, G.A.; Wojcik, E.M.; Nayar, R.; Savic-Prince, S.; Quek, M.L.; Kurtycz, D.F.; Rosenthal, D.L. The Paris System for Reporting Urinary Cytology: The Quest to Develop a Standardized Terminology. *Adv. Anat. Pathol.* **2016**, *23*, 193–201.
5. Barkan, G.A.; Wojcik, E.M.; Nayar, R.; Savic-Prince, S.; Quek, M.L.; Kurtycz, D.F.; Rosenthal, D.L. The Paris System for Reporting Urinary Cytology: The Quest to Develop a Standardized Terminology. *Acta. Cytol.* **2016**, *60*, 185–197.
6. Bochner, B.H.; Hansel, D.E.; Efstathiou, J.A.; Konety, B.; Lee, C.T.; McKiernan, J.M.; Plimack, E.R.; Reuter, V.E.; Sridhar, S.; Vikram, R.; et al. Urinary Bladder. In *AJCC Cancer Staging Manual*, 8th ed.; Amin, M.B.; Edge, S.B.; Greene, F.L.; Byrd, D.R.; Brookland, R.K.; Washington, M.K.; Gershenwald, J.E.; Compton, C.C.; Hess, K.R.; Sullivan, D.C.; et al.; Eds.; Springer: New York, USA, 2017; pp. 757–765.
7. Lee, C.H.; Chung, S.Y.; Moon, K.C.; Park, I.A.; Chung, Y.R.; Ryu, H.S. A Pilot Study Evaluating Fine-Needle Aspiration Cytology of Clear-Cell Renal Cell Carcinoma: Comparison of Ancillary Immunocytochemistry and Cytomorphological Characteristics of SurePath™ Liquid-Based Preparations with Conventional Smears. *Acta. Cytol.* **2015**, *59*, 239–247.
8. Lee, H.; Kim, K.; Woo, J.; Park, J.; Kim, H.; Lee, K.E.; Kim, H.; Kim, Y.; Moon, K.C.; Kim, J.Y.; et al. Quantitative Proteomic Analysis Identifies AHNAK (Neuroblast Differentiation-associated Protein AHNAK) as a Novel Candidate Biomarker for Bladder Urothelial Carcinoma Diagnosis by Liquid-based Cytology. *Mol. Cell. Proteomics.* **2018**, *17*, 1788–1802.
9. Han, D.; Moon, S.; Kim, Y.; Kim, J.; Jin, J.; Kim, Y. In-depth proteomic analysis of mouse microglia using a combination of FASP and StageTip-based, high pH, reversed-phase fractionation. *Proteomics.* **2013**, *13*, 2984–2988.
10. Rappsilber, J.; Mann, M.; Ishihama, Y. Protocol for micro-purification, enrichment, pre-fractionation and storage of peptides for proteomics using StageTips. *Nat. Protoc.* **2007**, *2*, 1896–1906.
11. Cox, J.; Neuhauser, N.; Michalski, A.; Scheltema, R.A.; Olsen, J.V.; Mann, M. Andromeda: a peptide search engine integrated into the MaxQuant environment. *J. Proteome. Res.* **2011**, *10*, 1794–1805.
12. Kim, J.Y.; Lee, H.; Woo, J.; Yue, W.; Kim, K.; Choi, S.; Jang, J.J.; Kim, Y.; Park, I.A.; Han, D.; et al. Reconstruction of pathway modification induced by nicotinamide using multi-omic network analyses in triple negative breast cancer. *Sci. Rep.* **2017**, *7*, 3466.
13. Baker, P.R.; Chalkley, R.J. MS-viewer: a web-based spectral viewer for proteomics results. *Mol. Cell. Proteomics.* **2014**, *13*, 1392–1396.
14. Kaimal, V.; Bardes, E.E.; Tabar, S.C.; Jegga, A.G.; Aronow, B.J. ToppCluster: a multiple gene list feature analyzer for comparative enrichment clustering and network-based dissection of biological systems. *Nucleic. Acids. Res.* **2010**, *38*, W96–W102.
15. Szklarczyk, D.; Gable, A.L.; Lyon, D.; Junge, A.; Wyder, S.; Huerta-Cepas, J.; Simonovic, M.; Doncheva, N.T.; Morris, J.H.; Bork, P.; et al. STRING v11: protein-protein association networks with increased coverage, supporting functional discovery in genome-wide experimental datasets. *Nucleic. Acids. Res.* **2019**, *47*, D607–D613.
16. Shannon, P.; Markiel, A.; Ozier, O.; Baliga, N.S.; Wang, J.T.; Ramage, D.; Amin, N.; Schwikowski, B.; Ideker, T. Cytoscape: a software environment for integrated models of biomolecular interaction networks. *Genome. Res.* **2003**, *13*, 2498–2504.
17. Tyanova, S.; Temu, T.; Sinitcyn, P.; Carlson, A.; Hein, M.Y.; Geiger, T.; Mann, M.; Cox, J. The Perseus computational platform for comprehensive analysis of (prote)omics data. *Nat. Methods.* **2016**, *13*, 731–740.

18. Carey, S.P.; Martin, K.E.; Reinhart-King, C.A. Three-dimensional collagen matrix induces a mechanosensitive invasive epithelial phenotype. *Sci. Rep.* **2017**, *7*, 42088.
19. Sanchez-Carbayo, M.; Socci, N.D.; Charytonowicz, E.; Lu, M.; Prystowsky, M.; Childs, G.; Cordon-Cardo, C. Molecular profiling of bladder cancer using cDNA microarrays: defining histogenesis and biological phenotypes. *Cancer. Res.* **2002**, *62*, 6973–6980.
20. Detre, S.; Saclani Jotti, G.; Dowsett, M. A "quickscore" method for immunohistochemical semiquantitation: validation for oestrogen receptor in breast carcinomas. *J. Clin. Pathol.* **1995**, *48*, 876–878.

2. Supplementary Figures:

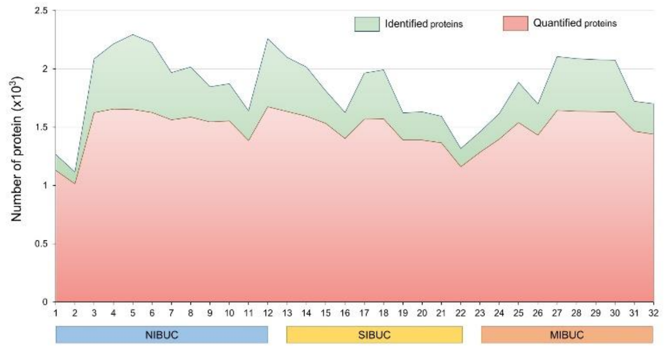

Figure S1. Identified and quantified proteins among 16 bladder urothelial carcinoma (BUC) liquid-based cytology (LBC) samples (duplicated analyses for each sample; 1-12, non-invasive BUC (NIBUC); 13-22, stromal-invasive BUC (SIBUC); 23-32, muscle-invasive BUC (MIBUC)).

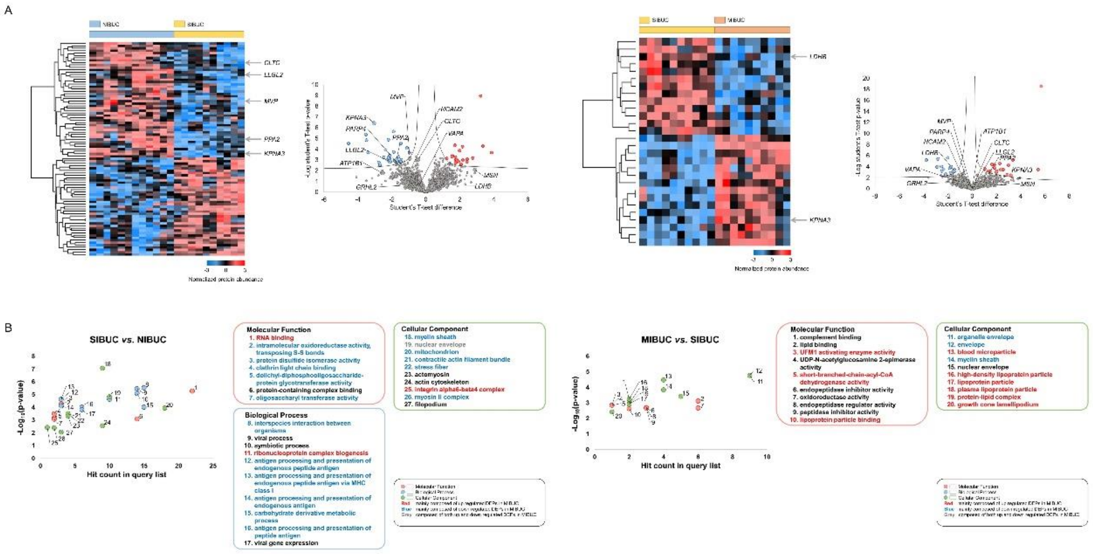

Figure S2. Hierarchical clustering and gene ontology results between two groups from bladder urothelial carcinoma (BUC) liquid-based cytology (LBC) samples (**A**) Hierarchical clustering and volcano plot between two groups from BUC LBC samples (left, stromal-invasive BUC (SIBUC) and non-invasive BUC (NIBUC); right, muscle-invasive BUC (MIBUC) and SIBUC). (**B**) gene ontology results between two groups from BUC LBC samples (left, SIBUC and NIBUC; right, MIBUC and SIBUC).

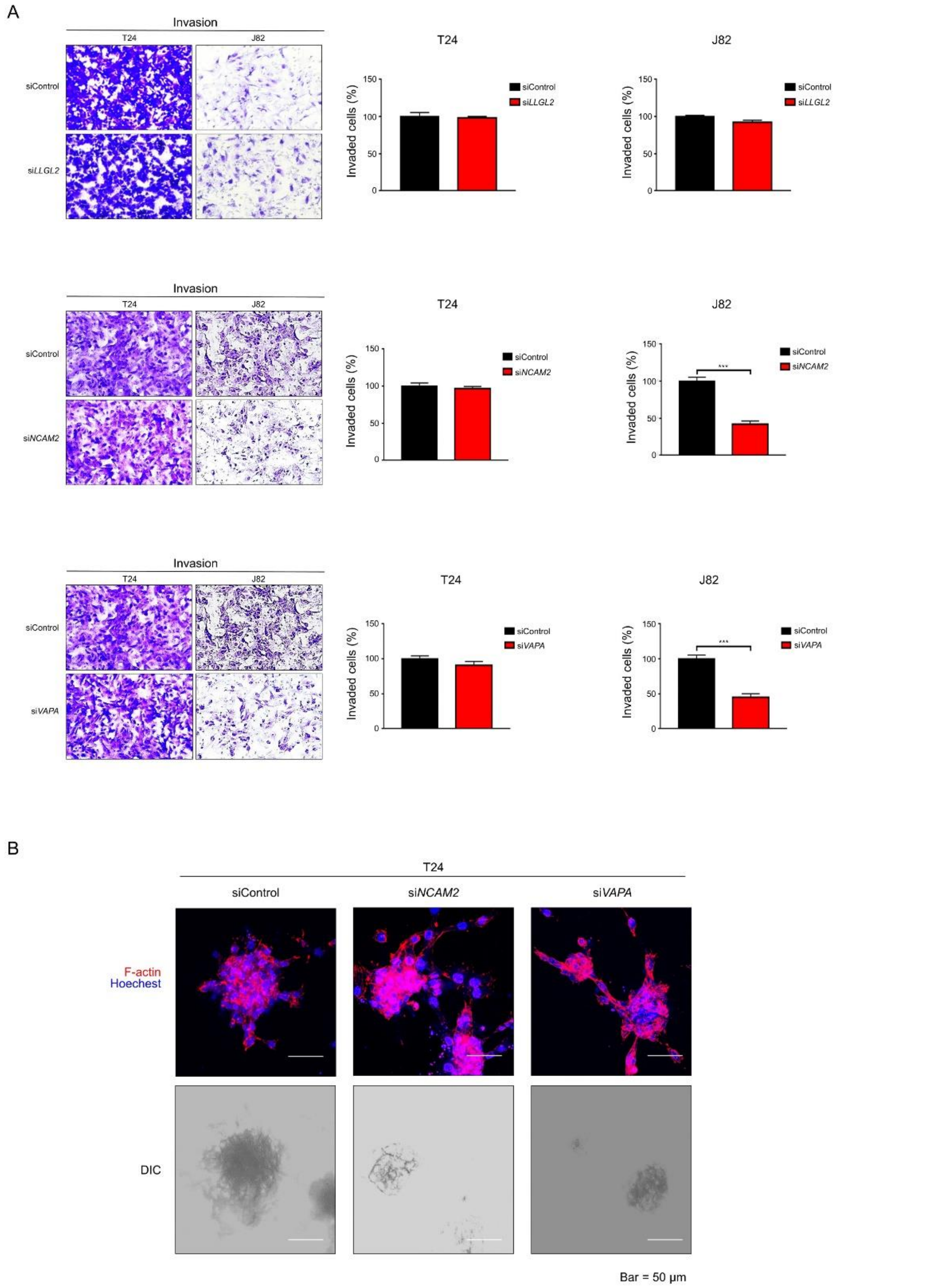

Figure S3. Functional validation of invasive role of *LLGL2*, *NCAM2*, and *VAPA* with small interfering RNA (siRNA) using two-dimensional (2D) and three-dimensional (3D) invasion assays. (A) 2D invasion assay (statistical significance, *** *p*-value < 0.001), (B) confocal microscope image of 3D dissemination.

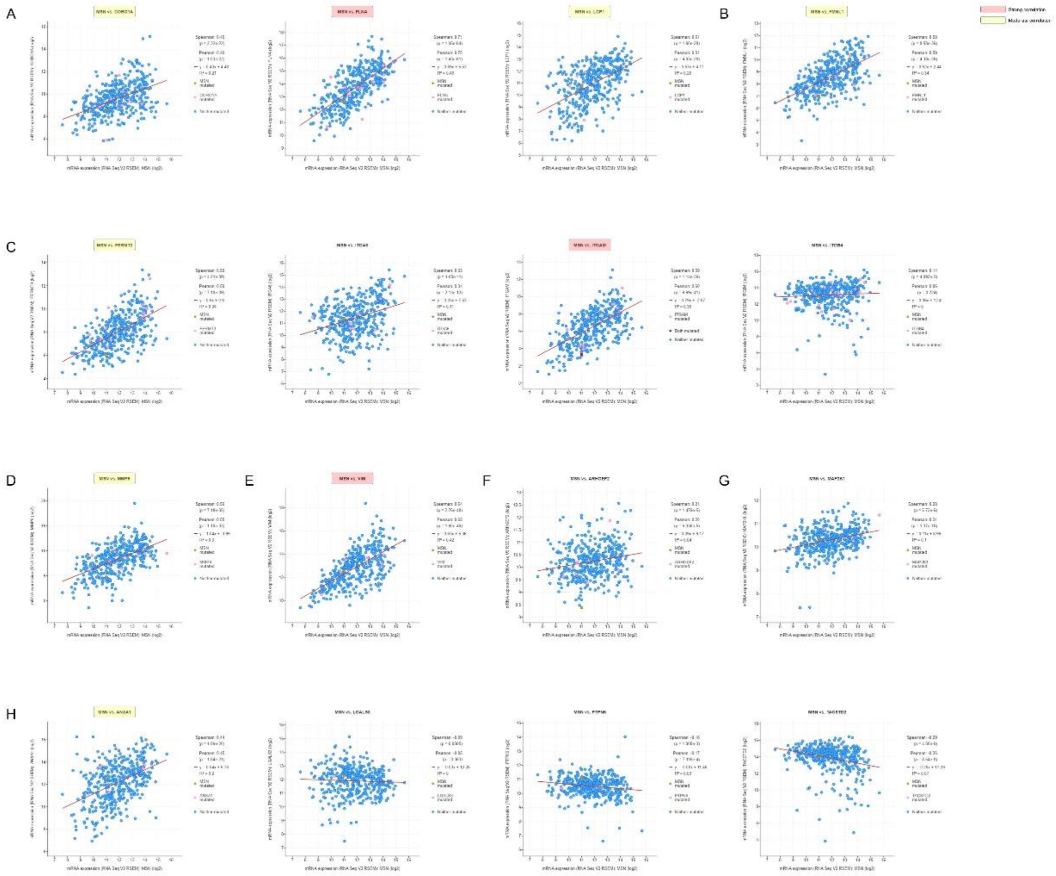

Figure S4. Co-expression analysis of *MSN* with key proteins in signaling pathway associated with cell motility. (A) Actin dynamics, (B) formin, (C) integrin signaling, (D) extracellular matrix (ECM) remodeling, (E) epithelial–mesenchymal transition (EMT) phenotype, (F) small GTPase activator, (G) MAPK pathway, (H) others.

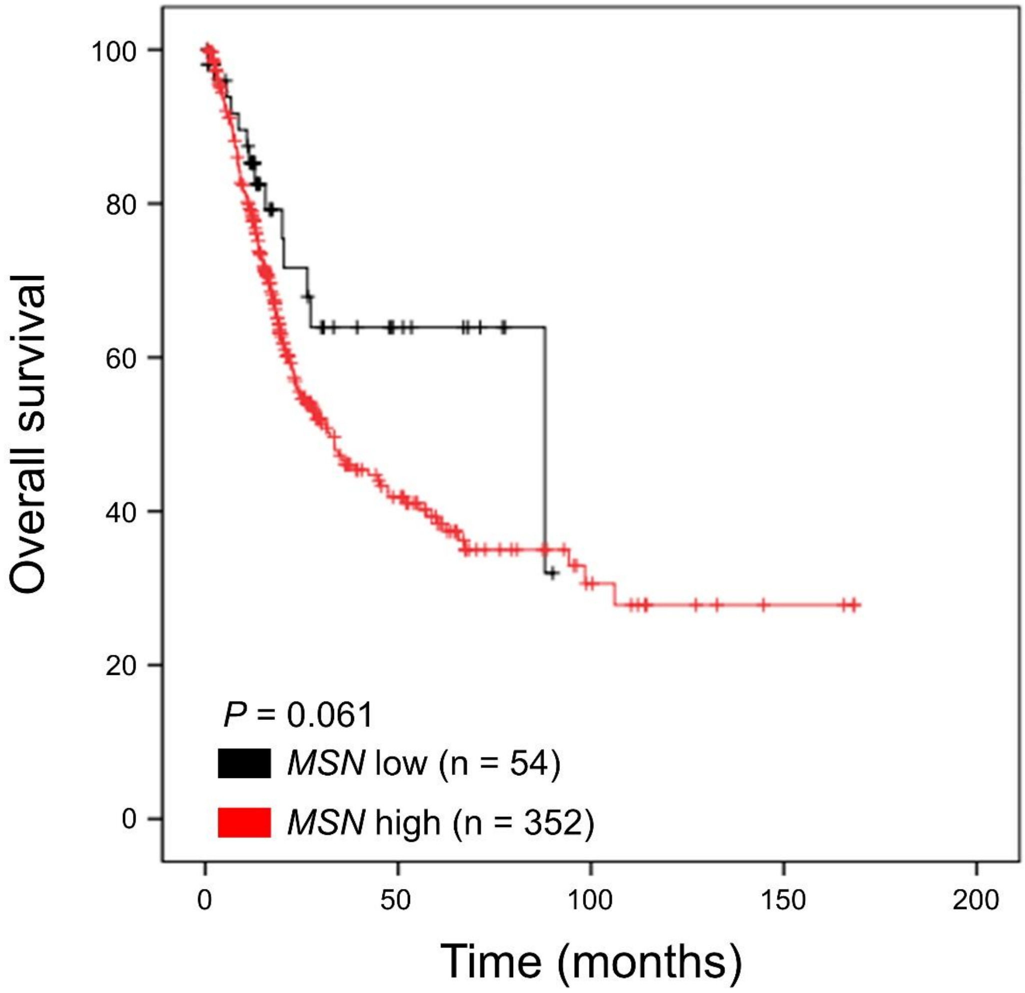

Figure S5. Kaplan–Meier survival curve for overall survival according to *MSN* RNA expression on The Cancer Genome Atlas (TCGA) public data.

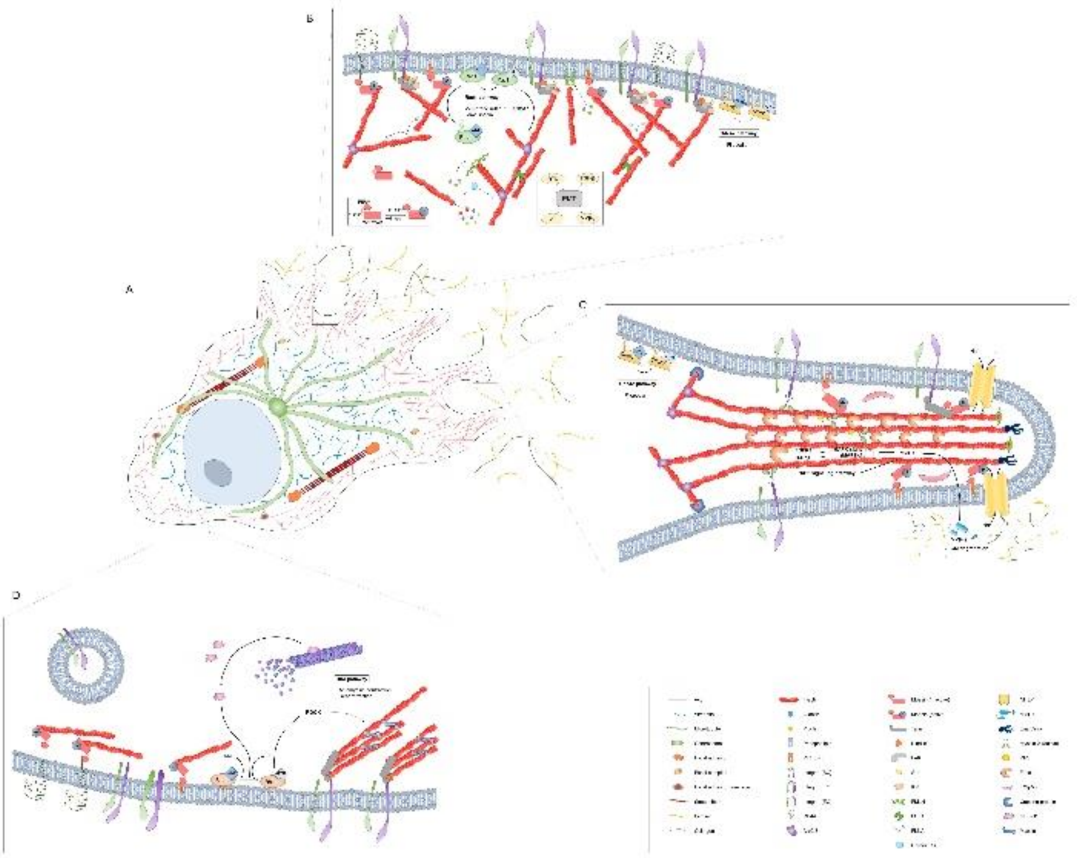

Figure S6. Proposed cellular aspect of bladder urothelial carcinoma (BUC) invasion process. (A) Overview of BUC invasion process, (B) lamellipodia formation, (C) filopodia formation, (D) rear retraction (abbreviations: C-ERMAD, C-terminal ERM-associated domain; ECM, extracellular matrix; FAK, focal adhesion kinase; FERM, 4.1-band ERM; FGFR3, fibroblast growth factor receptor 3; GDP, guanosine diphosphate; GTP, guanosine triphosphate; ILK, integrin-linked kinase; integrin (BC, bent-closed; EC, extended-closed; EO, extended-open); MAPK, mitogen-activated protein kinase; MMPs, matrix metalloproteinases; $PI(4,5)P_2$, phosphatidylinositol 4,5-bisphosphate; ROCK, Rho-associated protein kinase; RTK, receptor tyrosine kinase; TGF-β, transforming growth factor β; VT, vimentin).

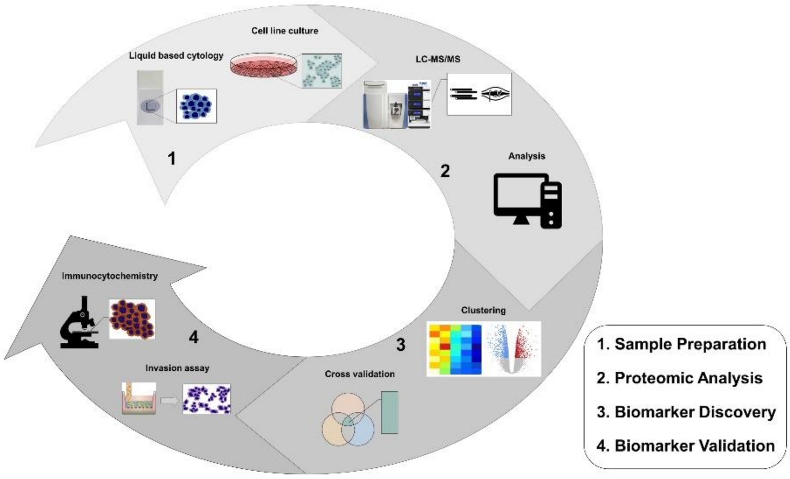

Figure S7. Overall workflow of the study design. Proteomic analysis of bladder urothelial carcinoma (BUC) liquid-based cytology (LBC) samples and cell line samples with invasion assay and immunocytochemistry (ICC) validation.
